# Crucial role of craftsmanship spirit in fostering innovative behavior among skilled talents in the manufacturing sector

**DOI:** 10.3389/fpsyg.2024.1407426

**Published:** 2025-01-07

**Authors:** Yuzhu Li, Qiuyue Zhang, Wenyin Yang

**Affiliations:** College of Business, Beijing Wuzi University, Beijing, China

**Keywords:** craftsmanship spirit, innovative behavior, innovative climate, innovative self-efficacy, knowledge sharing

## Abstract

**Introduction:**

Innovation remains pivotal for establishing and sustaining a competitive edge within the manufacturing industry. Skilled talents play a vital role in driving innovation and entrepreneurship in this sector. Their creative prowess significantly determines the success of a nation’s innovation-centric development strategy. Craftsmanship spirit emerges as an indispensable mental fortitude for exceptionally skilled talents.

**Methods:**

Drawing on social cognitive theory and social exchange theory, this study constructed a theoretical model to explore the intricate relationship between craftsmanship spirit and the innovative behavior of skilled talents, which was subsequently validated through empirical research.

**Results:**

This study showed that craftsmanship spirit actively fostered the innovative behavior of skilled talents. This facilitation was mediated through two interconnected variables: innovative self-efficacy and knowledge sharing among skilled talents, with organizational innovation climate assuming a nuanced regulatory role.

**Discussion:**

Consequently, enterprises are encouraged to nurture craftsmanship spirit, amplify innovative self-efficacy, promote knowledge sharing, and proactively instill an innovative climate within their organization. These measures can collectively inspire and amplify innovative behavior among skilled talents.

## Introduction

Innovation has always been a key driver in establishing and maintaining a competitive edge within the manufacturing industry. Skilled talents serve as the cornerstone of innovation and entrepreneurship in manufacturing. Also, their level of creativity directly shapes the success of the innovation-driven development strategy of a nation, thereby impacting the competitiveness and future trajectory of the nation. Individual creativity is a complex interplay of various factors. Although research has delved into areas such as education and family environment, universities continue to refine their talent development environments; however, the outcomes remain suboptimal (Guo and Deng, 2020). The reasons for this challenge are twofold. First, intrinsic factors are fundamental to personal development, whereas extrinsic factors provide supportive conditions. Education and family environments do not consistently and effectively enhance the creativity of skilled talents (Liu, 2017), as the creative potential of skilled talents is fundamentally determined by their intrinsic factors. Second, the work environment of skilled talents compared with education and family environment substantially influences their creativity. Therefore, delving into the inherent traits of skilled talents and their work environment is crucial.

Among the intrinsic characteristics of skilled talents, the craftsmanship spirit emerges as a pivotal source of inspiration for innovation and entrepreneurship in manufacturing powerhouses such as Germany and Japan. The primary goal of craftsmanship spirit is to craft industry-leading products of the highest quality, surpassing those of competitors. Achieving this objective requires skilled talents to be guided by the spirit of craftsmanship in their innovative behavior. Research suggests that this guidance occurs through both cognitive and behavioral dimensions.

First, craftsmanship spirit can be internalized as an intrinsic quality within skilled talents. Second, it can be externalized as the pursuit of high-quality work outcomes by each skilled talent. According to social cognitive theory, the cognitive aspect, particularly innovation self-efficacy, closely aligns with the creative behavior itself. Individuals with high innovation self-efficacy exhibit increased focus, persistence, patience, and a strong belief in their capacity to achieve innovative outcomes (Gu and Peng, 2011). According to social exchange theory, employees committed to excellence in their work processes often engage in explicit or implicit knowledge exchange between individuals and organizations, fostering willingness to share knowledge. This willingness, in turn, enhances opportunities for collective learning and progress, thereby increasing the likelihood of innovation.

Therefore, this study used craftsmanship spirit as the independent variable to investigate its impact on the innovative behavior of skilled talents. It examined two key mediating factors: the cognitive aspect of skilled talents—innovation self-efficacy—and the attitudinal aspect—knowledge-sharing willingness.

Regarding the work environment, skilled talents are continually engaged in social exchange relationships with their organizations. The behavioral responses of skilled talents are, to a certain extent, influenced and constrained by these exchange relationships. The research results also confirm that high-level exchange relationships prompt employees to engage in more high-quality behaviors (Liu et al., 2020). According to social exchange theory, when skilled talents engage in effective communication, learning, and exchange, they are likely to be influenced by the rich innovative climate provided by the organization. In turn, they may exhibit more positive behaviors, potentially leading to increased innovative behavior. Hence, this study posited a moderating role of the innovative climate in the mechanism through which craftsmanship spirit influences the innovative behavior of skilled talents. The findings of this study enrich the empirical understanding of how craftsmanship spirit impacts employee behavior, and the antecedent of the key factors influencing innovative behavior, and the mechanisms that reinforce innovative behavior in skilled talents.

## Literature review and research hypotheses

### Literature review

#### Skilled talents

Skilled talents generally refer to individuals in frontline operational roles in production, manufacturing, or service sectors who use their technical skills to create tangible products or value. Guo et al. (2017) supplemented the definition of skilled personnel by highlighting their crucial role in regional industrial development and technological product updates. These individuals, knowledge-based employees, translate the designs of research and development personnel into tangible products. Building upon the aforementioned definition, this study incorporates the explanation of skilled talents provided by Shen and Pan (2009). It defines skilled personnel as individuals with specific professional knowledge and skills, who create tangible value in roles such as production and manufacturing.

#### Craftsmanship spirit

Research on the craftsmanship spirit by scholars predominantly encompasses macro and theoretical explorations. It focuses on tracing historical origins, defining its conceptual connotations, examining its cultivation and development, and analyzing the factors that influence it. Specifically, concerning the tracing of historical origins, the emergence of the craftsmanship spirit in China can be traced back to the reign of Emperor Shun, with documented references to “artisans” appearing since then. The connection between artisans and the craftsmanship spirit has become increasingly intimate with progressive improvements in societal and economic levels. Artisans not only embody the craftsmanship spirit but also actively contribute to its creation (Zhang, 2019). For instance, China’s achievements in porcelain craftsmanship, silk production, inventions, and innovations have captivated the world, showcasing the profound, distinctive, and vibrant craftsmanship spirit cultivated by Chinese artisans.

Regarding the definition of conceptual connotations, Liu et al. (2022) proposed that the craftsmanship spirit arises as a conscious behavior in the professional process, rooted in exquisite craftsmanship and manifested through a relentless pursuit of excellence and professional responsibility. According to this conceptual delineation, the craftsmanship spirit embodies professional behavior, representing the high-level expertise of individuals in their respective fields and their pursuit of mission-driven values in their work. Zhao et al. (2020) suggested that the craftsmanship spirit reflects individuals’ specific work values in their current jobs, reflecting various work objectives they passionately strive for.

Regarding the outcome variables of the craftsmanship spirit, existing research confirms its significant positive impact on innovation performance (Peng and Yang, 2022), and product quality (Senge, 2006) at the enterprise level. Additionally, the craftsmanship spirit plays a significant role in proactive behaviors (Gao, 2022) and job satisfaction (Li et al., 2021) at the employee level.

Existing research has rarely delved into the role of the craftsmanship spirit in innovative behaviors, leaving room for exploration in this study.

#### Innovative behaviors

Employees are increasingly recognized as a significant source of organizational innovation and, therefore, scholars interpret individual innovative behaviors from various perspectives. From the standpoint of employee innovation ideology, Hurt et al. (1977) argued that individual innovative behavior falls within the realm of ideology, representing a willingness possessed by innovative individuals, manifested in “new perspectives generated by individuals in organizational work” and “behavior manifested in practice.” From the perspective of the employee innovation process, Scott and Bruce (1994) defined employee innovative behavior as the sum of processes and activities from generating new ideas to implementing them. Kleysen and Street (2001) suggested that this process included exploring innovative thinking, creating new ideas, seeking support, and facilitating implementation. Wang and Chang (2017) believed that the individuals promoting new ideas in their work deserve special emphasis, apart from the innovative ideas themselves, in the phased process of innovation.

The research on the influence of innovative behavior can be summarized into two main categories: the impact of intrinsic factors of employees and external situational factors on innovative behavior. The influence of intrinsic factors of employees on innovative behavior mainly involves personal traits (Liu et al., 2020), employees’ human capital (Guo et al., 2017), self-awareness and efficacy (Edmondson and Lei, 2014), knowledge-sharing behavior (Zhang et al., 2020), and so forth. Regarding the influence of external situational factors on innovative behavior, previous research focused more on leadership styles (Yang and Yang, 2020) and organizational atmosphere and support (Ren and Zhang, 2015).

The literature analysis revealed that scholars conducted extensive research on the antecedents of the spirit of craftsmanship; however, data on the posterior variables were lacking. Also, the impact of the spirit of craftsmanship on employees’ innovative behavior remains unexplored, leaving room for the development of this study. Based on the research group of skilled talents, the spirit of craftsmanship represents high standards, strict requirements, and increasingly refined work values of this group. Therefore, examining the relationship of craftsmanship with the innovative self-efficacy and knowledge-sharing behavior of skilled talents is of great significance. At the same time, this can further improve the mechanism of the relationship between the spirit of craftsmanship and the innovative behavior of skilled talents. Employee innovative behavior is also influenced by external factors, among which organizational innovation atmosphere is an important aspect. However, scholars’ findings on organizational innovation atmosphere as a moderating variable are somewhat insufficient. This study helped enrich the research results on organizational innovation atmosphere serving as a moderating variable.

### Research hypotheses

#### Craftsmanship spirit positively influences the innovative behavior of skilled talents

Craftsmanship spirit, as an inherent driving force for employee innovative behavior (Li et al., 2018), instills skilled talents with passion in their work and encourages them to pursue innovation (Ye et al., 2018). It emphasizes the continuous learning required of artisans, cultivating a mindset conducive to innovation and internalizing the spirit of innovation into the work behavior of artisans, including changes in products and processes (Su and Wang, 2018). The craftsmanship spirit can inspire artisans to meticulously create products and deliver services (Liu, 2017). It is an important variable of employee occupational characteristics, primarily reflected in the accumulation of work, flexible thinking, and skill enhancement, driving the occurrence of innovative behavior during work processes (Cheng and Tian, 2016). Therefore, the following hypothesis is proposed:

*H1: Craftsmanship spirit positively influences the innovative behavior of skilled talents.*

#### Mediating role of innovative self-efficacy

Innovative self-efficacy refers to the strong belief of individuals in their ability to engage in innovative workplace behaviors (Li et al., 2020). Craftsmanship spirit represents not only tradition but also persistence and innovation. The driving force behind its creation stems from the craftsman’s strong self-perception. The interaction between craftsmanship spirit and innovative self-efficacy can be transformed into the values of “artisan” or the confidence strength of “craftsmanship spirit.” Craftsmanship spirit essentially reflects employees’ work beliefs and behavioral guidelines, constituting a higher-order synthesis of individual values.

Based on social cognitive theory, previous studies have found that higher levels of innovative self-efficacy can enhance the willingness of individuals to innovate, effectively stimulating their innovative behavior; conversely, it often leads to a lack of belief among employees, resulting in a negative attitude toward innovative activities (Wang et al., 2016). Innovative individuals often face challenges or doubts from various sources when generating creative ideas or transforming them into actual behaviors. At such times, if individuals have sufficient confidence in themselves, their innovative behavior is more likely to succeed (Yang et al., 2011). Strong innovative self-efficacy can guide technical workers to actively integrate explicit skills and transform them into enthusiasm for their own unique knowledge. In this process, a sense of achievement arises from the continuous improvement of skills, ultimately leading to surpassing others (Tierney and Farmer, 2002). Therefore, the following hypotheses are proposed:

*H2: Craftsmanship spirit positively impacts the innovative self-efficacy of skilled talents.*

*H3: Innovative self-efficacy positively impacts the innovative behavior of skilled talents.*

*H4: Innovative self-efficacy mediates the relationship between craftsmanship spirit and innovative behavior of skilled talents.*

#### Mediating role of knowledge sharing

Zhu et al. (2021) pointed out that craftsmanship spirit, as an excellent professional spirit, might prompt employees to take responsibility proactively, actively provide better suggestions and strategies for work and the company, and facilitate the emergence of behaviors such as offering advice and organizational citizenship. Zhao et al. (2020) revealed that craftsmanship spirit might reflect employees’ established work norms, which can guide their work behavior preferences. The motivation in craftsmanship spirit has a self-directed effect (Guo and Deng, 2020).

Innovation is inseparable from knowledge. According to Scott and Bruce’s explanation of innovative behavior, individuals need to communicate their ideas with others and seek supporters and alliances while generating innovative behavior. Knowledge sharing is necessary for generating innovative behavior (Obrenovic et al., 2020). In knowledge sharing, organizational members increase opportunities for mutual learning and cooperation, thereby increasing the possibility of innovation (Tsai, 2009). Therefore, the following hypotheses are proposed:

*H5: Craftsmanship spirit has a positive impact on knowledge sharing.*

*H6: Knowledge sharing positively impacts the innovative behavior of skilled talents.*

*H7: Knowledge sharing mediates the relationship between craftsmanship spirit and innovative behavior of skilled talents.*

#### Innovative self-efficacy and knowledge sharing—intricate variables with significant interconnections

Innovative self-efficacy positively influences knowledge sharing. Studies focusing on organizations and employees reveal that the key factors influencing knowledge sharing stem from individual beliefs. When employees perceive themselves as capable of sharing valuable organizational knowledge, they are typically more motivated to share their insights with colleagues (Lin, 2007). Furthermore, when employees believe they stand to gain—whether through organizational rewards or the fulfillment of helping others—they may cultivate a more positive attitude toward knowledge sharing (Van Den Hooff and De Ridder, 2004). Sun et al. (2012) empirically investigated the relationship between innovative self-efficacy and knowledge sharing using structural equation modeling.

The craftsmanship spirit enhances employees’ innovative self-efficacy, which, in turn, positively influences their innovative behaviors. Additionally, this spirit fosters knowledge sharing among employees, while knowledge sharing enhance innovative behaviors. Therefore, given the role of innovative self-efficacy in promoting knowledge sharing, we propose the following hypothesis:

*H8: Innovative self-efficacy and knowledge sharing act as a chain-mediating mechanism between craftsmanship spirit and employee innovation, where craftsmanship spirit encourages knowledge sharing through innovative self-efficacy, thereby facilitating employee innovation.*

**Figure 1:**
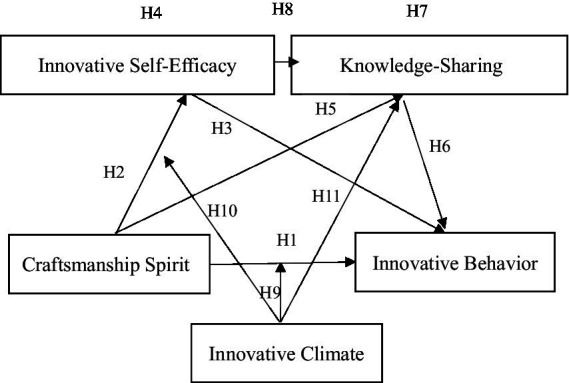
The theoretical model.

Based on the research hypothesis, a theoretical model is established, as shown in Figure 1.

#### Moderating role of innovative climate

Innovative climate represents the subjective psychological perception of skilled talents toward the innovative environment, indicating, to some extent, the level of support for innovation provided by leadership and organizations (Ahmed, 1998). Social cognitive theory emphasizes the interaction between the environment, individual cognition, and behavior. Within this theoretical framework, the innovative climate is considered as a social environment whereas craftsmanship spirit and innovation self-efficacy are perceived by individuals as cognitive aspects. Knowledge-sharing and innovative behavior arise from the interaction between the social environment and cognitive processes.

An innovative climate fosters a social environment rich in opportunities for innovation and learning. In such an environment, individuals can observe the craftsmanship spirit, knowledge sharing, and innovative behaviors of others. Also, they can transform these observations into their own behaviors and attitudes through observational learning. The knowledge-sharing and innovative behaviors of individuals may be encouraged, recognized, and rewarded in an organizational environment with an innovative climate. Such support and encouragement can significantly enhance the innovation self-efficacy of individuals, which is the belief in their ability to implement innovative actions. This self-efficacy is conducive to the more effective transformation of craftsmanship spirit into actual innovative behavior and increases the likelihood of individuals engaging in knowledge sharing to drive innovation. Positive social support and team interactions within the innovative climate can facilitate knowledge sharing, cooperation, and collaborative innovation, creating a more favorable environment for effectively transforming craftsmanship spirit into innovative behavior.

Based on the aforementioned theoretical considerations and analysis, the following hypotheses are proposed:

*H9: The innovative climate moderates the relationship between craftsmanship spirit and the innovative behavior of skilled talents.*

*H10: The innovative climate moderates the relationship between craftsmanship spirit and the innovation self-efficacy of skilled talents.*

*H11: The innovative climate moderates the relationship between knowledge-sharing and innovative behavior of skilled talents.*

## Methods

### Sample and procedure

From November 2022 to August 2023, a questionnaire survey was conducted on skilled talents in the Chinese manufacturing industry based on the principles of convenience and randomness. We distributed and collected survey questionnaires through the Credamo platform. Skilled talents are those who engage in technical and skilled work in frontline positions. The scope of manufacturing industry is restricted through the Credamo platform, mainly including 31 industries such as agricultural and sideline food processing industry, food manufacturing industry, alcohol, beverage, and refined manufacturing industry, tobacco products industry, textile industry, textile and clothing industry, and so forth. We initially distributed questionnaires on a small scale to ensure the accuracy of the data, eliminating ineligible ones. We expanded the survey on a larger scale after ensuring the reliability and validity of the questionnaire. A total of 468 questionnaire forms were distributed, and 400 valid responses were obtained after filtering based on mandatory questions and response time, resulting in a validity rate of 85.47% (see Figure 1).

### Measures

Scales widely used both domestically and internationally were selected for this study. The content of each variable was compared through a review of relevant literature, and the most suitable items for this study were selected. A 5-point Likert-type scale (1 = completely disagree to 5 = completely agree) was used for all measures.

#### Craftsmanship spirit

This study employed the craftsmanship spirit scale developed by Zhao et al. (2020). The scale divided craftsmanship spirit into 5 dimensions: personal growth, responsibility, excellence, reputation, and commitment, including 20 items such as “I constantly explore my potential in my work” (*α* =0.89). See the attachment for the specific scale.

#### Innovative behavior

Compared with foreign scales, the employee innovative behavior scale compiled by Chinese scholar Zhang Zhengang is more suitable for the Chinese context and aligns better with the Chinese way of thinking. Therefore, this study used this scale, comprising eight items such as “*I often seek opportunities to improve work methods and processes*” (*α* = 0.95). See the attachment for the specific scale.

#### Innovative self-efficacy

This study adopted the measurement scale based on the scale developed by scholars such as Tierney and Farmer (2002), which is widely accepted and used. The scale included four items such as “*I feel confident in coming up with novel ideas*” (*α* = 0.85). See the attachment for the specific scale.

#### Knowledge sharing

This study focused on examining the overall level of employees’ knowledge sharing. Therefore, we chose the scale revised by Lu et al. (2006) based on the Bock and Kim scale, including eight items such as “*In my daily work, I actively share business knowledge with colleagues*” (*α* = 0.94). See the attachment for the specific scale.

#### Innovation climate

This study used the revised keys scale by Liu and Shi (2009), including 12 items such as “*At work, my colleagues are willing to share their methods and techniques with each other*” (*α* = 0.93). See the attachment for the specific scale.

## Results

### Data reliability and validity analysis

#### Common method bias test

All items from the five scales of craftsmanship spirit, innovative behavior, innovative self-efficacy, knowledge sharing, and innovation climate were analyzed using SPSS 26.0. The results are presented in Table 1. Nine eigenvalues were greater than 1, and the variance explained by the largest factor without rotation was 32.67%, which was below the reference threshold of 40%. This indicated that the common method bias in the sample data of this study was acceptable. Therefore, the data analysis based on this was considered reliable.

**Table 1:** Total variance explained.

Component	Initial eigenvalues			Sum of squared loadings		
	Total	Percentage of variance	Cumulative percentage	Total	Percentage of variance	Cumulative percentage
1	16.986	32.666	32.666	16.986	32.666	32.666
2	6.005	11.548	44.215	6.005	11.548	44.215
3	3.677	7.072	51.287	3.677	7.072	51.287
4	2.529	4.864	56.151	2.529	4.864	56.151
5	1.791	3.445	59.596	1.791	3.445	59.596
6	1.593	3.063	62.658	1.593	3.063	62.658
7	1.426	2.743	65.401	1.426	2.743	65.401
8	1.227	2.360	67.761	1.227	2.360	67.761
9	1.128	2.169	69.929	1.128	2.169	69.929

### Validity analysis using AMOS software

Single-factor, two-factor, three-factor, four-factor, and five-factor analysis models were constructed for the analysis data, and each model was individually tested. The results are presented in Table 2. For the five-factor model, *χ*^2^/DF = 2.994 < 3, IFI = 0.932 > 0.9, TLI = 0.924 > 0.9, CFI = 0.931 > 0.9, and RMSEA = 0.071 < 0.08. These values indicated that the model had the best fit, significantly outperforming the fit of the four-factor, three-factor, two-factor, and one-factor models. It suggested good discriminant validity among the five variables. The questionnaire design in this study appeared to be reasonable.

**Table 2:** Results of confirmatory factor analysis.

Model	χ^2^/DF	IFI	TLI	CFI	RMSEA
Five-factor CS + ISE + KS + IC + IB	2.994	0.932	0.924	0.931	0.071
Four-factor CS, ISE + KS + IC + IB	5.444	0.846	0.830	0.845	0.106
Three-factor CS, ISE, KS + IC + IB	7.995	0.755	0.732	0.754	0.132
Two-factor CS, ISE, KS, IC + IB	8.998	0.718	0.694	0.717	0.142
Single-factor CS, ISE, KS, IC, IB	14.009	0.540	0.502	0.539	0.181

CS is craftsmanship spirit, ISE is Innovative Self-Efficacy, KS is Knowledge Sharing, IC is Innovative Climate, IB is Innovative Behavior.

### Descriptive analyses

Table 3 presents the descriptive statistics and correlations among the variables.

**Table 3:** Descriptive statistical results of samples.

Project	Option	Frequency	Percentage	Cumulative percentage
Gender	male	280	70.00	70.00
	female	120	30.00	100.00
Age	20–30 years	83	20.75	20.75
	30–39 years	230	57.50	78.25
	40–49 years	55	13.75	92.00
	50–59 years	25	6.25	98.25
	60 years and above	7	1.75	100.00
Education	Junior high school and below	13	3.25	3.25
	high school	23	5.75	9.00
	Technical secondary school	26	6.50	15.50
	junior college	60	15.00	30.50
	undergraduate	269	67.25	97.75
	master	7	1.75	99.50
	doctor	2	0.50	100.00
Length of service	One year and below	9	2.25	2.25
	1–5 years	62	15.50	17.75
	5–10 years	229	57.25	75.00
	10 year and above	100	25.00	100.00
Technical level	Junior technical workers	212	53.00	53.00
	Intermediate skilled worker	90	22.50	75.50
	Senior technical worker	48	12.00	87.5
	technician	13	3.25	90.75
	Senior technician	12	3.00	93.75
	No evaluation	25	6.25	100.00
Total		400	100.00	100.00

### Correlation analysis

Correlation analysis was conducted on various variables using SPSS 26.0, and the results are presented in Table 4. The findings indicated a significant positive correlation between craftsmanship spirit and innovative behavior, with a correlation coefficient of 0.292 (*p* < 0.01). Craftsmanship spirit was also significantly positively correlated with innovative self-efficacy (correlation coefficient = 0.311, *p* < 0.01) and knowledge sharing (correlation coefficient = 0.346, *p* < 0.01). Additionally, innovative self-efficacy and innovative behavior exhibited a significant positive correlation (correlation coefficient = 0.529, *p* < 0.01), as did knowledge-sharing and innovative behavior (correlation coefficient = 0.514, *p* < 0.01). The correlation coefficient between innovative self-efficacy and knowledge sharing was 0.555 (*p* < 0.01), indicating a significant positive correlation. These results provided preliminary validation of the research hypotheses 1, 2, 3, 5, and 6 and established a foundation for testing other hypotheses.

**Table 4:** Sample correlation analysis results.

Variables	Average value	Standard ation	Gender	Age	Education	Length of service	Technical level	CS	IB	ISE	KS	IA
Gender	1.700	0.459	1									
Age	2.107	0.862	−0.299**	1								
Education	4.615	1.267	−0.018	0.040	1							
Length of service	3.013	1.202	0.220**	−0.103*	0.248**	1						
Technical level	2.413	1.379	−0.129**	0.389**	−0.095	−0.172**	1					
CS	4.122	0.537	0.054	0.021	0.045	0.095	−0.161**	1				
IB	4.006	0.873	0.090	−0.068	0.189**	0.346**	−0.175**	0.292**	1			
ISE	4.027	0.928	0.082	0.025	0.132**	0.334**	−0.095	0.311**	0.529**	1		
KS	4.253	0.789	0.120*	−0.024	0.138**	0.350**	−0.148**	0.346**	0.514**	0.555**	1	
IA	4.239	0.605	0.000	0.011	0.146**	0.380**	−0.157**	0.289**	0.501**	0.544**	0.755**	1

*p < 0.05, **p < 0.01.

### Hypothesis testing

Building upon the earlier analyses, this study incorporated sex, age, education level, years of work experience, and technical proficiency as control variables in a hierarchical regression analysis. This was done to validate the positive impact of craftsmanship spirit on innovative behavior, examine the mediating roles of innovative self-efficacy and knowledge sharing between craftsmanship spirit and innovative behavior. The moderating effects of innovation climate on the relationships between craftsmanship spirit and innovative behavior, craftsmanship spirit and innovative self-efficacy, and knowledge-sharing and innovative behavior were explored.

#### Primary effects test

Control variables such as sex and age were initially included in the regression analysis for validating primary effects, forming Model 1. Subsequently, the independent variable craftsmanship spirit was introduced, forming Model 2. The results of the analysis are presented in Table 5.

**Table 5:** Regression analysis results of craftsmanship spirit on innovative behavior.

Variables	IB	
	Model 1	Model 2
Gender	0.014	0.001
Age	0.007	−0.022
Education	0.105*	0.103
Length of service	0.298***	0.282***
Technical level	−0.115*	−0.068
CS		0.250***
R^2^	0.144	0.203
△R^2^	0.133	0.191
F	13.204***	16.711***

n = 400. *p < 0.05, **p < 0.01, ***p < 0.001.

As shown in Table 5, the *R*^2^ value of Model 1 was 0.144, indicating that sex, age, education level, years of work experience, and technical proficiency collectively explained 14.4% of the variance in innovative behavior. Following an *F*-test for Model 1, an *F* value of 13.204 (*p* < 0.001) was obtained. The regression coefficients for education level, years of work experience, and technical proficiency were 0.105 (*p* < 0.05), 0.298 (*p* < 0.001), and − 0.115 (*p* < 0.05), respectively. This suggested a positive impact of education level and years of work experience and a negative impact of technical proficiency on innovative behavior.

Upon introducing the independent variable craftsmanship spirit in Model 2, the *F* value increased to 16.711 (*p* < 0.001), and the *R*^2^ value increased from 0.144 to 0.203. The regression coefficient (*β*) for craftsmanship spirit was 0.250 (*p* < 0.001), indicating a significant positive correlation with innovative behavior. Thus, Hypothesis 1 was supported.

### Mediation effects test

For the mediation effects test of innovative self-efficacy, control variables such as sex and age were initially included in hierarchical regression analyses, forming Models 1 and 3. These models were used to examine the relationships between control variables and innovative self-efficacy, as well as the relationships between control variables and innovative behavior. Based on Model 1, craftsmanship spirit was introduced in hierarchical regression analysis to form Model 2, examining the relationship between craftsmanship spirit and innovative self-efficacy. Furthermore, based on Model 1, innovative self-efficacy was included in hierarchical regression analysis to form Model 5, examining the relationship between innovative self-efficacy and innovative behavior. Lastly, based on Model 2, innovative self-efficacy was included in hierarchical regression analysis to form Model 6, examining the mediating role of innovative self-efficacy between craftsmanship spirit and innovative behavior. The specific results of the hierarchical regression analysis are presented in Table 6.

**Table 6:** Mediation effects test for innovative self-efficacy.

Variables	ISE		IB			
	Model 1	Model 2	Model 3	Model 4	Model 5	Model 6
Gender	0.033	0.018	0.014	0.001	−0.001	−0.007
Age	0.091	0.059	0.007	−0.022	−0.035	−0.047
Education	0.045	0.043	0.105	0.103	0.084	0.085*
Length of service	0.313***	0.296***	0.298***	0.282***	0.154**	0.158**
Technical level	−0.068	−0.016	−0.115	−0.068	−0.083	−0.061
CS		0.276***		0.250***		0.134**
ISE					0.460***	0.420***
R^2^	0.122	0.195	0.144	0.203	0.329	0.345
△R^2^	0.111	0.183	0.133	0.191	0.319	0.333
F	10.939	15.865	13.204	16.711	32.147	29.491

*n* = 400. *p < 0.05, **p < 0.01, ***p < 0.001.

As shown in Table 6, in Model 1, years of work experience positively influenced innovative self-efficacy (*β* = 0.313, *p* < 0.001). In Model 2, craftsmanship spirit positively influenced innovative self-efficacy (*β* = 0.276, *p* < 0.001), validating Hypothesis 2. In Model 5, innovative self-efficacy positively influenced innovative behavior (*β* = 0.460, *p* < 0.001), validating Hypothesis 3. In Model 6, when both craftsmanship spirit and innovative self-efficacy were included in hierarchical regression analysis for innovative behavior, the regression coefficient of craftsmanship spirit changed from 0.250 to 0.134. However, innovative self-efficacy continued to significantly and positively influence innovative behavior (*β* = 0.420, *p* < 0.001), validating Hypothesis 4.

Further validation of the mediating role of innovative self-efficacy was conducted using the bootstrap method, as shown in Table 7. For the mediating role of innovative self-efficacy between craftsmanship spirit and innovative behavior, the 95% confidence interval was 0.088–0.223, excluding 0. Additionally, the mediating effect of innovative self-efficacy accounted for 51.783%. Hypothesis 4 was further supported.

**Table 7:** Mediation effects test for knowledge sharing.

Variables	KSB		IB			
	Model 1	Model 2	Model 3	Model 4	Model 5	Model 6
Gender	0.057	0.041	0.014	0.001	−0.011	−0.015
Age	0.064	0.029	0.007	−0.022	−0.022	−0.034
Education	0.048	0.045	0.105	0.103	0.084	0.085
Length of service	0.314***	0.295***	0.298***	0.282***	0.160**	0.166***
Technical level	−0.107	−0.050	−0.115	−0.068	−0.068	−0.048
CS		0.305***		0.250***		0.130**
KS					0.437***	0.393***
R^2^	0.138	0.227	0.144	0.203	0.308	0.323
△R^2^	0.127	0.215	0.133	0.191	0.298	0.311
F	12.569	19.204	13.204	16.711	29.207	26.701

*n* = 400. *p < 0.05, **p < 0.01, ***p < 0.001.

For the mediation effects test of knowledge sharing, a hierarchical regression analysis was conducted following the methodology employed for the innovative self-efficacy mediation effects test. The specific analysis results are presented in Table 7.

As shown in Table 7, in Model 1, years of work experience positively influenced knowledge sharing (*β* = 0.314, *p* < 0.001). In Model 2, craftsmanship spirit positively influenced knowledge sharing (*β* = 0.305, *p* < 0.001), validating Hypothesis 5. In Model 5, knowledge sharing positively influenced innovative behavior (*β* = 0.437, *p* < 0.001), validating Hypothesis 6. In Model 6, when both craftsmanship spirit and knowledge sharing were included in hierarchical regression analysis for innovative behavior, the regression coefficient of craftsmanship spirit changed from 0.250 to 0.393. However, knowledge sharing continued to significantly and positively influence innovative behavior (*β* = 0.130, *p* < 0.001), validating Hypothesis 7.

#### Chain mediation effects testing

This study constructed a chain mediation model to examine the parallel mediation effects of innovative self-efficacy and knowledge sharing. This study used the bootstrap method to ensure the accuracy of the test. Before analyzing the data, 5,000 sampling repetitions with a 95% confidence interval needed to be set. The specific analysis results are shown in Table 8.

**Table 8:** Mediation effects testing results (Bootstrap Method).

	Effect value	95% confidence intervals	
		Lower limit	Upper limit
Path 1: CS → ISE → IB	0.171	0.055	0.166
Path 2: CS → KS → IB	0.093	0.022	0.106
Path 3: CS → ISE → KS → IB	0.075	0.020	0.079
Total Effect	0.474	0.321	0.627

*n = 400.*

As shown in Table 8, in Path 1: craftsmanship spirit → innovative self-efficacy → innovative behavior, the effect size was 0.171, with a 95% confidence interval of 0.055–0.166. This confidence interval did not include 0, confirming Hypothesis 4 once again. In Path 2: craftsmanship spirit → knowledge sharing → innovative behavior, the effect size was 0.093, with a 95% confidence interval of 0.022–0.106. This confidence interval also did not include 0, confirming Hypothesis 7 once again. In Path 3: craftsmanship spirit → innovative self-efficacy → knowledge sharing→ innovative behavior, the effect size was 0.075, with a 95% confidence interval of 0.020–0.079. In summary, innovative self-efficacy and knowledge sharing mediated the relationship between craftsmanship spirit and innovative behavior, validating the chain mediation model.

#### Moderation effects test

First, the moderation effects of the innovation climate on the relationship between knowledge-sharing and innovative behavior were examined. The hierarchical regression analysis was conducted on the centralized processed knowledge-sharing and innovative climate concerning innovative behavior, resulting in Model 2. Subsequently, the interaction term between knowledge sharing and the innovation climate was incorporated into the hierarchy regression analysis of innovative behavior based on Model 2, resulting in Model 3. The specific analysis results are presented in Table 9.

**Table 9:** Moderation effect test of the innovation climate between knowledge sharing and innovation behavior.

Variables	Model 1	Model 2	Model 3
	IB		
Control Variable	Control	Control	Control
KS	0.437***	0.282***	0.342***
IC		0.219**	0.306***
KS × IC			0.168*
R^2^	0.308	0.328	0.339
△R^2^	0.298	0.315	0.325
F	29.207***	27.272***	25.022***

*n* = 400. *p < 0.05, **p < 0.01, ***p < 0.001.

As shown in Table 9, in Model 3, the Δ*R*^2^ value was 0.325 and the *F* value was 25.022 (*p* < 0.001), indicating a well-fitted hierarchical regression result. Additionally, the regression coefficient of the interaction term between knowledge sharing and the innovation climate in Model 3 was 0.168 (*p* < 0.05). This suggested the significance of the interaction term, affirming that the innovation climate positively moderated the relationship between knowledge-sharing and innovative behavior, thus validating Hypothesis 9.

**Figure 2:**
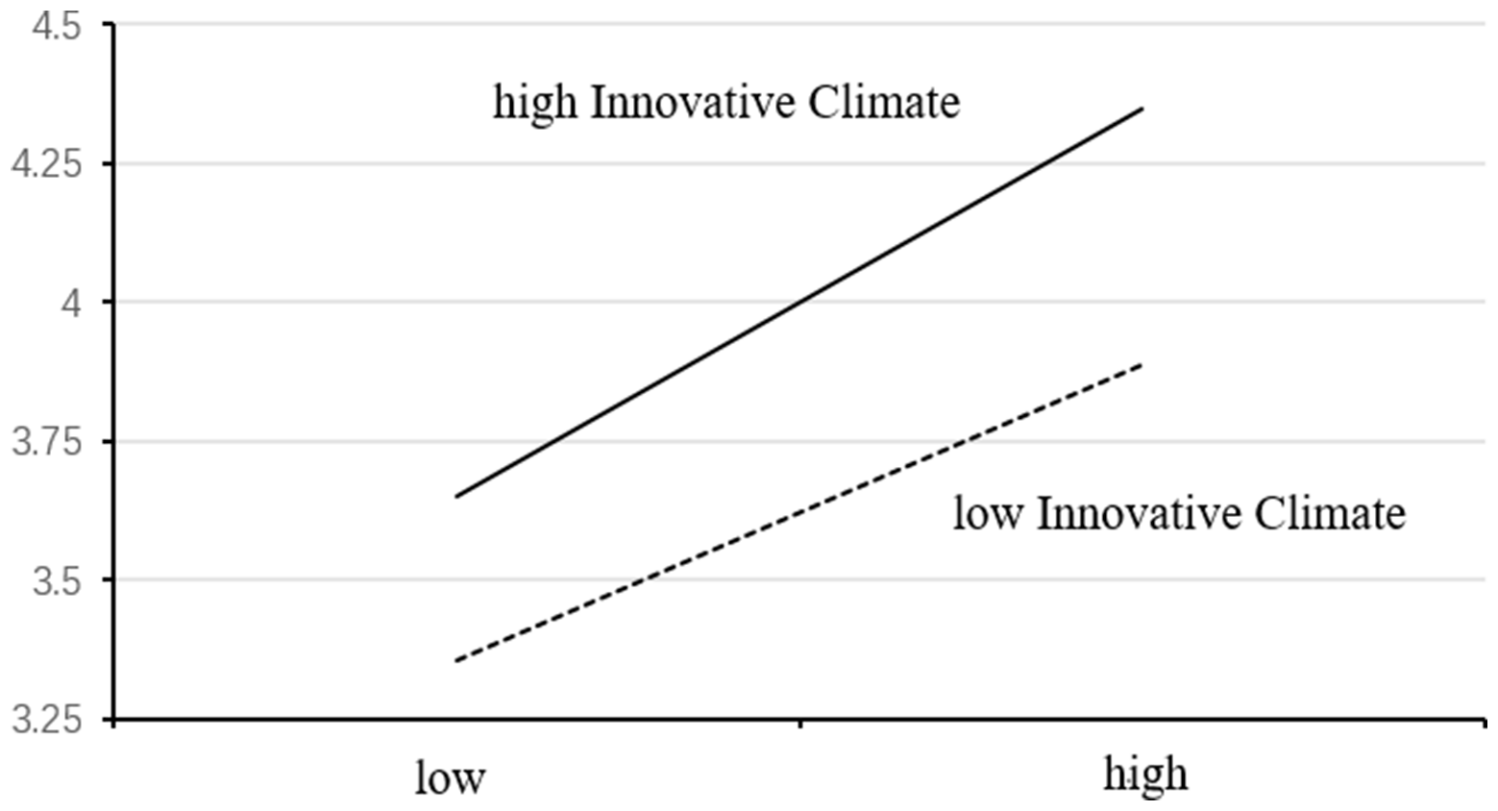
The moderating effect of innovation climate on knowledge sharing and innovation behavior.

A simple slope analysis was conducted to provide a more intuitive analysis of the moderating effect of the innovation climate. As illustrated in Figure 2, the slope value of the effect line from knowledge sharing to innovative behavior was higher in a high-innovation climate compared with a low-innovation climate. This implied that knowledge sharing is more effective in promoting innovative behavior in a situation with a pronounced innovation climate. Therefore, the innovation climate positively moderated the relationship between knowledge-sharing and innovative behavior, confirming Hypothesis 9 once again.

We employed the same testing method that was applied to examine the moderating effects of the innovation climate on the relationship between knowledge-sharing and innovative behavior to test the moderating effects of the innovation climate on the following relationships: the relationship between craftsmanship spirit and innovative self-efficacy (Table 10) and the relationship between craftsmanship spirit and innovative behavior (Table 11).

**Table 10:** Testing the moderating effect of innovative climate on the relationship between craftsmanship spirit and innovative behavior.

Variables	Model 1	Model 2	Model 3
	IB		
Control variable	Control	Control	Control
CS	0.000**	0.000**	0.006**
IA		0.000**	0.000**
CS*IA			0.000**
*R* ^2^	0.085	0.275	0.301
*F*	36.980***	75.110***	56.769***
△*R*^2^	0.085	0.189	0.026

*n* = 400. *p < 0.05, **p < 0.01, ***p < 0.001.

**Table 11:** Analysis of the moderating effect of innovative climate on the relationship between craftsmanship spirit and innovative self-efficacy.

Variables	Model 1	Model 2	Model 3
	IS		
Control variable	Control	Control	Control
CS	0.000**	0.000**	0.001**
IA		0.000**	0.000**
CS*IA			0.005**
*R* ^2^	0.097	0.322	0.336
调整*R*^2^	0.094	0.319	0.330
*F* 值	42.621	94.343	66.651
△*R*^2^	0.097	0.225	0.013

*n* = 400. *p < 0.05, **p<0.01, ***p<0.001.

## Conclusions and implications

### Research conclusions

The craftsmanship spirit of technical talents in the manufacturing industry significantly influences their innovative behavior. Exploring its impact mechanism reveals that knowledge sharing and self-efficacy serve as chain mediators in the relationship between craftsmanship spirit and innovative behavior. Additionally, the innovation climate moderates the relationships between craftsmanship spirit and innovative behavior, craftsmanship spirit and innovative self-efficacy, as well as knowledge-sharing and innovative behavior. The research findings unveil novel influencing factors for innovative behavior, elucidating the impact mechanism of craftsmanship spirit on innovative behavior. This has significant reference value for fostering and motivating innovative behavior among technical talents.

### Management insights

The findings of this study underscore the significance of craftsmanship spirit, innovative self-efficacy, knowledge sharing, and innovation climate for skilled talents in the manufacturing industry, as well as their shaping and stimulating roles in fostering innovative behavior among these talents. They provide management insights for manufacturing organizations seeking to enhance innovative behavior among their skilled talents.

#### Cultivating craftsmanship spirit in skilled talents

Craftsmanship spirit is recognized as a crucial source of inspiration for innovation and entrepreneurship in manufacturing powerhouses such as Germany and Japan. The conclusions drawn in this study also affirm the pivotal impact of craftsmanship spirit on innovative behavior among skilled talents in the manufacturing industry. Therefore, manufacturing organizations should prioritize and cultivate the craftsmanship spirit in their skilled talents.

Craftsmanship spirit emphasizes the mastery of practical skills and the accumulation of hands-on experience. Companies should provide skilled talents with specialized education and skill training courses, including technical training and industry certification programs, ensuring a profound knowledge base and exceptional technical skills in specific domains. Establishing a mentorship program is also recommended, allowing experienced artisans to impart their accumulated skills and experiences to the next generation of skilled talents. This enables the new generation to collaboratively solve real technical challenges and adapt to new demands through close cooperation with mentors.

Implementing incentive mechanisms, such as rewards, encourages skilled talents to demonstrate craftsmanship spirit in their practical work, thereby providing additional motivation, such as monetary incentives or honorary titles. Cultivating a company culture emphasizing skills and practicality is essential, as the values within the culture can encourage skilled talents to embody the craftsmanship spirit, thus showcasing enthusiasm for their work and a commitment to excellence.

#### Enhancing innovative self-efficacy of skilled talents

The innovative self-efficacy of skilled talents can effectively promote their innovative behavior and transform their craftsmanship spirit into tangible innovative outcomes. Therefore, manufacturing companies should prioritize the enhancement of innovative self-efficacy among skilled talents. Innovative self-efficacy reflects the confidence of skilled talents in their own innovative capabilities. Companies are advised to provide opportunities for skilled talents to practice innovation, allowing them to gain practical experience in successful innovation.

This can be achieved by encouraging skilled talents to participate in innovation projects, solve real-world problems, or propose technical and skill-related improvement suggestions. Recognizing and rewarding innovative ideas and solutions put forward by skilled talents can help establish a stronger sense of innovative self-confidence. Ensuring that skilled talents have sufficient support in terms of resources, including time, technical equipment, and training resources, is crucial to enabling them to fully unleash their innovation potential.

Stimulating interest in innovation involves creating a culture fostering curiosity and enthusiasm. Companies can achieve this by showcasing successful innovation cases, inviting industry experts to share their experiences, and using other means to spark the interest and passion of skilled talents in innovation.

#### Encouraging knowledge sharing in skilled talents

Elevating the knowledge sharing of skilled talents is crucial for organizations seeking to foster a craftsmanship spirit among employees and promote innovation. Companies should cultivate an open, transparent, and inclusive communication environment to encourage knowledge sharing among skilled talents, allowing them the freedom to express their opinions and share knowledge.

Establishing knowledge-sharing platforms, such as internal social networks, team collaboration platforms, professional communities, or groups, can facilitate the easy sharing of documents, experiences, and best practices among skilled talents. This enhances the accessibility and share ability of information, enabling skilled talents to share knowledge effortlessly. Additionally, creating platforms specifically focused on certain themes or projects encourages the transfer of knowledge and facilitates collaboration.

Supporting knowledge sharing within workflow processes is essential. Integrating knowledge sharing into workflow processes, for example, encouraging the sharing of experiences and knowledge during project reviews or regular meetings, ensures that knowledge sharing becomes an integral part of daily work.

#### Fostering an organizational culture of innovation

The culture of innovation plays a pivotal, multifaceted role and serves as a crucial task for businesses aiming to enhance employee creativity. Promoting a culture of innovation can stimulate creative thinking among employees, foster the emergence of new ideas, and propel the organization toward continuous progress. It can enhance the positive impact of craftsmanship spirit on the innovative behavior and innovative self-efficacy of skilled talents, as well as amplify the positive influence of knowledge sharing on innovative behavior. Therefore, manufacturing companies should actively cultivate an innovative climate.

Senior leadership should actively support innovation, recognizing it as a key factor for organizational success. The encouragement and exemplary role of leaders are vital in establishing an innovative climate. Implementing incentive mechanisms, such as distributing bonuses, awards, and promotions, encourages employees who propose innovative ideas or successfully implement innovative projects.

Facilitating the formation of diverse teams within the organization, comprising individuals with different backgrounds, expertise, and experiences, can contribute to the generation of innovative thoughts from various perspectives. Creating a culture that encourages innovation attempts is essential. Encouraging employees to experiment with new approaches while embracing and learning from failures allows employees to draw lessons from setbacks, continuously improve, and cultivate a culture characterized by innovation and flexibility. This culture provides employees with more opportunities to unleash their creativity and achieve innovation.

### Implications

#### Theoretical implications

First, this study further explored the empirical research mechanisms through which craftsmanship spirit influenced employee behavior.

Second, it revealed the empirical research mechanisms of antecedent variables for innovative behavior. The study mainly delved into individual cognition and behavior, elucidating and validating the dual mediating role of innovative self-efficacy and knowledge sharing in the relationship between craftsmanship spirit and innovative behavior. This comprehensive understanding of the mechanisms broadened the scope of the impact mechanism of craftsmanship spirit while enriching the research on the antecedent variables of innovative behavior.

Third, it uncovered the reinforcement mechanism of innovative behavior among skilled talents. Based on social exchange theory, the study identified the innovation climate as a boundary condition for the impact of knowledge sharing on innovative behavior among skilled talents. This study is significant for providing guidance to organizations on incentivizing innovative behavior among skilled talents.

#### Practical implications

First, skilled talents are a crucial force supporting the development of the manufacturing industry, and employee innovative behavior is the inexhaustible driving force for the sustainable development of manufacturing enterprises. Therefore, exploring the impact of craftsmanship spirit on the innovative behavior of skilled talents is conducive to the transformation, upgrading, and sustained development of manufacturing enterprises.

Second, the discovery that craftsmanship spirit influences the innovative behavior of skilled talents through innovative self-efficacy and knowledge sharing is novel. For manufacturing enterprises, this finding provides new insights and references for promoting the transformation of craftsmanship spirit into innovative behavior at the employee level, thereby injecting new impetus into the development of manufacturing enterprises. For managers, this discovery helps implement relevant management measures to maintain the innovative self-efficacy of skilled talents, such as increasing employee incentives and improving assessment systems. Simultaneously, it encourages managers to prioritize the effective use of knowledge resources among skilled talents and provide the necessary conditions for sharing. This approach enhances the innovative behavior of skilled talents and ultimately boosts the overall innovative behavior of the manufacturing industry.

Third, from an organizational perspective, the moderating effects of the innovation climate prompt manufacturing enterprises to pay more attention to the working environment and atmosphere of employees, thus fully leveraging the multiple moderating effects of the innovation climate.

### Limitations and future research

The data for variables in this study were obtained through self-assessment by survey respondents within the same timeframe. Consequently, a potential risk of common method bias existed. Although this study passed the common method bias test and we tried our best to minimize its effects, the possibility of some impact still exists. Therefore, future studies should consider examining and analyzing data from different sources. In our preliminary research and design, the innovation climate played a moderating role between craftsmanship spirit and knowledge sharing of employees. However, this was not supported by the data collected. This might be attributed to the fact that the amount of data we collected was not large enough. Therefore, we plan to address this issue or identify the root cause of the issue in the future.

Craftsmanship spirit is a crucial source of inspiration for innovation and entrepreneurship in manufacturing powerhouses. This study focused solely on investigating the impact and influencing mechanisms of craftsmanship spirit on the innovative behavior of skilled talents. Our future studies will emphasize on researching outcome variables related to craftsmanship spirit, expanding the scope of craftsmanship spirit research, and providing insights for actively and fully harnessing the power of craftsmanship spirit.

Innovation is a key driver for creating and maintaining competitive advantages in enterprises. This study primarily explored the impact mechanism of craftsmanship spirit on innovative behavior. Future studies can promote the investigation of other factors influencing innovative behavior, thus broadening the understanding of the multifaceted aspects contributing to innovation.

We should acknowledge that, despite the robustness of the conducted analyses, the findings were based on self-reported data collected at a single point in time. Thus, future research should consider using data from diverse sources and time points to mitigate potential common method bias and provide a more comprehensive understanding of the studied relationships. Additionally, expanding the scope of research to include diverse outcome variables related to craftsmanship spirit and exploring the influence of various factors on innovative behavior can contribute to a more holistic understanding of these complex dynamics.

## Data Availability

The raw data supporting the conclusions of this article will be made available by the authors, without undue reservation.
