# Supplementary material for: Crucial role of craftsmanship spirit in fostering innovative behavior among skilled talents in the manufacturing sector
Source: Front Psychol. 2025 Jan 7;15:1407426. doi: 10.3389/fpsyg.2024.1407426 (PMC11745889; doi:10.3389/fpsyg.2024.1407426)
Supplement: Supplementary file 1 [file Table_1.DOCX]

Appendix

**Craftsmanship Spirit Scale**

| Dimensionality | Number | Item |
| --- | --- | --- |
| Personal growth | 1 | Constantly explore your potential at work |
|  | 2 | Continuous improvement in the process of completing their own work |
|  | 3 | Keep improving your job skills |
|  | 4 | Continuously improve your business expertise at work |
| responsibility | 5 | Take responsibility for the work I've done |
|  | 6 | See your job as a responsibility and commitment |
|  | 7 | Be serious about what I do |
|  | 8 | It is my duty to finish the work with high quality |
| excellence | 9 | Strive for perfection in your work details |
|  | 10 | Keep thinking about how to do your job better |
|  | 11 | Try to avoid defects or deficiencies in your work |
|  | 12 | Set a higher standard for yourself than the organization requires |
| reputation | 13 | If my work is not done well, it will make me feel dishonorabl |
|  | 14 | My personal reputation depends on how well my work is done |
|  | 15 | Let people know that something was done by me |
|  | 16 | The quality of your work is also a reflection of your character |
| commitment | 17 | There is no rush to see results in the short term |
|  | 18 | Do only one job in your life |
|  | 19 | Adhere to their own standards, not be swayed by the outside world |
|  | 20 | See work as a career, not just a tool to make money |

**Innovative Behavior Scale**

| Number | Item |
| --- | --- |
| 1 | I am always looking for opportunities to improve my working methods and processes |
| 2 | I often try new ways to solve problems at work |
| 3 | I often think about things from different angles |
| 4 | I will not miss any opportunity to understand and discover the problem |
| 5 | I often suggest introducing new working methods in the company |
| 6 | I often take risks to support new ideas or ideas |
| 7 | I often introduce some new working methods to my colleagues |
| 8 | I often test the effectiveness of new working methods |

**Innovative Self-Efficacy** **Scale**

| Number | Item |
| --- | --- |
| 1 | I think I'm good at coming up with novel ideas |
| 2 | I have confidence in my ability to solve problems creatively |
| 3 | I have a knack for further complementing other people's ideas |
| 4 | I'm good at finding new ways to solve problems |

**Knowledge Sharing scale**

| Number | Item |
| --- | --- |
| 1 | In my daily work, I actively share my business knowledge with my colleagues. |
| 2 | I keep my own work experience to myself and never share it easily with others |
| 3 | I share my useful work experience and insights with everyone |
| 4 | After learning new knowledge that is useful for work, I promote it so that more people can learn it |
| 5 | Unless the company asks me to, I generally do not share my work experience and skills with others |
| 6 | On my job, I take out my knowledge and share it with more people |
| 7 | I actively use the existing information technology resources of the company to share my knowledge |
| 8 | As long as other colleagues in the company need it, I always speak without reservation and without holding back |

**Innovative Climate Scale**

| Number | Item |
| --- | --- |
|  | At work, my colleagues will support and assist each other |
|  | At work, my colleagues are willing to share each other's methods and techniques |
|  | My colleagues often communicate and discuss problems in their work |
|  | When I have new ideas, my colleagues actively express their opinions and suggestions |
|  | My supervisor respects and tolerates different opinions and objections from subordinates |
|  | My supervisor encourages subordinates to make proposals to improve production or service |
|  | My supervisor will support and assist the subordinates to realize the creative ideas in the work |
|  | My supervisor is a great example of innovation |
|  | The company encourages employees to try new things and learn from mistakes |
|  | The company appreciates and recognizes employees with innovative and enterprising spirit |
|  | Companies often reward employees for their innovative ideas |
|  | The company advocates freedom, openness and innovation |
